# Erythema elevatum diutinum – An atypical case presentation exposing the histopathological progression of disease

**DOI:** 10.1177/2050313X251343268

**Published:** 2025-05-23

**Authors:** Mina Youakim, Ahmed Shah, Lynne H. Robertson

**Affiliations:** 1 Cumming School of Medicine, University of Calgary, AB, Canada; 2 Department of Pathology and Laboratory Medicine, University of Calgary, AB, Canada; 3 Department of Laboratory Medicine and Pathology, Mayo Clinic, Rochester, MN, USA; 4 Division of Dermatology, Department of Medicine, University of Calgary, AB, Canada

**Keywords:** erythema elevatum diutinum, leukocytoclastic vasculitis, palpable pupura

## Abstract

Erythema elevatum diutinum is a distinct and rare form of chronic, recurrent leukocytoclastic vasculitis that typically presents with firm, smooth erythematous to violaceous papules or nodules symmetrically distributed over the extensor surfaces. Herein, we report an unusual case of erythema elevatum diutinum in a 32-year-old female who presented with a several-year history of chronic recurrent palpable purpura prior to the development of classic nodular lesions. This presentation facilitated a timely diagnosis and highlighted the histopathologic progression of the disease broadening the clinical spectrum of erythema elevatum diutinum.

## Introduction

Erythema elevatum diutinum (EED) is a distinct and rare form of chronic, recurrent leukocytoclastic vasculitis (LCV), with only several 100 cases described in the literature.^1^ The condition typically presents with firm, smooth erythematous to violaceous papules or nodules symmetrically distributed over the extensor surfaces and may pose a diagnostic challenge for physicians. Herein, we report an unusual case of EED in a 32-year-old female which manifested initially as chronic recurrent palpable purpura prior to the development of classic nodular lesions of EED. This unique presentation facilitated a timely diagnosis of EED, highlighted the histopathologic progression of the disease, and broadened the clinical spectrum of EED.

## Case presentation

**Figure 1.**
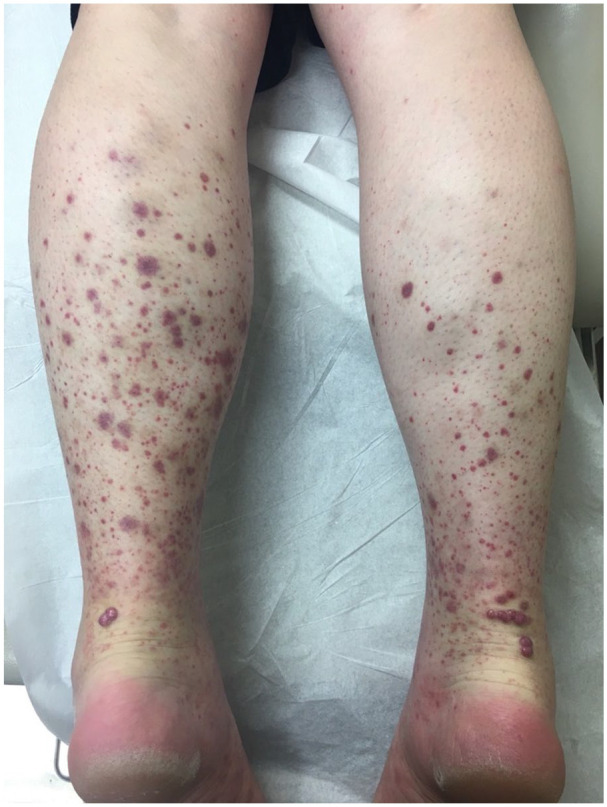
Maculopapular purpura on the lower legs bilaterally. Violaceous, firm, dome-shaped papules over the Achilles area bilaterally.

A 32-year-old female presented with a 3-year history of recurrent violaceous macules and papules on the lower legs. These typically resolved spontaneously over several weeks and recurred every 3–4 months. They were occasionally pruritic but otherwise asymptomatic, and the patient had no associated systemic symptoms. One year prior to presentation, the patient noted the development of asymptomatic and persistent nodular lesions on the lower legs and knees following each episode. Initial examination revealed petechia, purpuric macules, and palpable purpura of the lower extremities, and several smooth, firm, violaceous papules over the posterior ankles (Figure 1).

**Figure 2.**
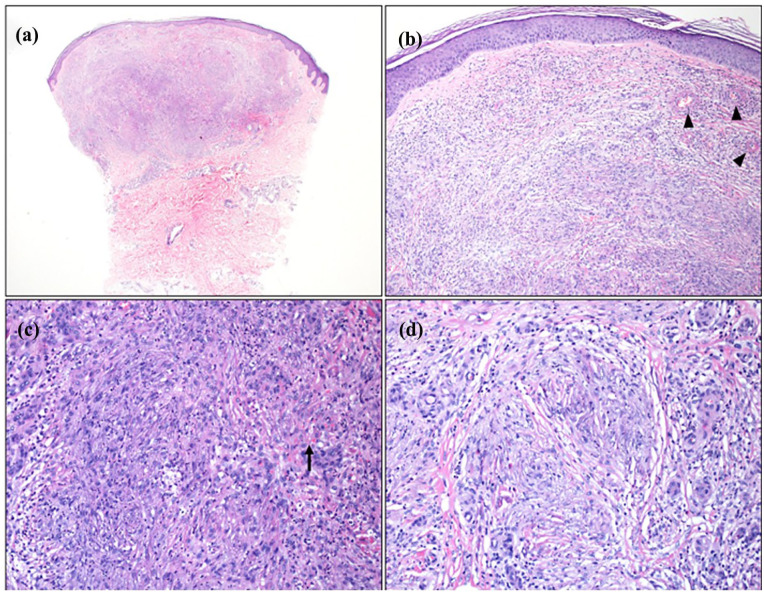
Representative histopathological images of right anterior ankle biopsy specimen. (a) Scanning magnification showing dense nodular infiltrate involving the superficial and mid-reticular dermis (H&E, ×20). (b) Nodular capillary proliferation (granulation tissue) associated with perivascular and interstitial neutrophilic infiltrate, features of leukocytoclastic vasculitis including fibrinoid necrosis of blood vessel walls (highlighted by arrowheads), and storiform dermal fibrosis (H&E, ×100). (c) Capillary proliferation with dense neutrophilic infiltrate, erythrocyte extravasation (highlighted by arrow), and background storiform pattern of dermal fibrosis (H&E, ×200). (d) Onion-skinning fibrosis around the vessels with scattered neutrophils, lymphocytes, and focal eosinophils (H&E, ×200).

A biopsy from the palpable purpura showed changes consistent with an LCV. The histopathology from a biopsy of one of the firm papules demonstrated a nodular proliferation of vessels within the dermis with multiple foci of perivascular neutrophilic infiltrate with fibrinoid necrosis of the blood vessel walls and surrounding leukocytoclasis, in keeping with LCV. These changes occurred on a background of granulation tissue and scarring, with some areas exhibiting a storiform pattern (Figure 2). Histopathologic special stains for infectious organisms were negative.

The patient’s clinical and histopathologic findings were consistent with a diagnosis of EED. Additional lab investigations including a complete blood count, serum chemistry, antinuclear antibody and extractable nuclear antigen, rheumatoid factor, antineutrophil cytoplasmic antibodies, serum protein electrophoresis, urinalysis, human immunodeficiency virus, hepatitis B and C screen, serologic tests for syphilis, and immunoglobulin A transglutaminase were normal. A chest X-ray and ophthalmological evaluation revealed no abnormalities.

**Figure 3.**
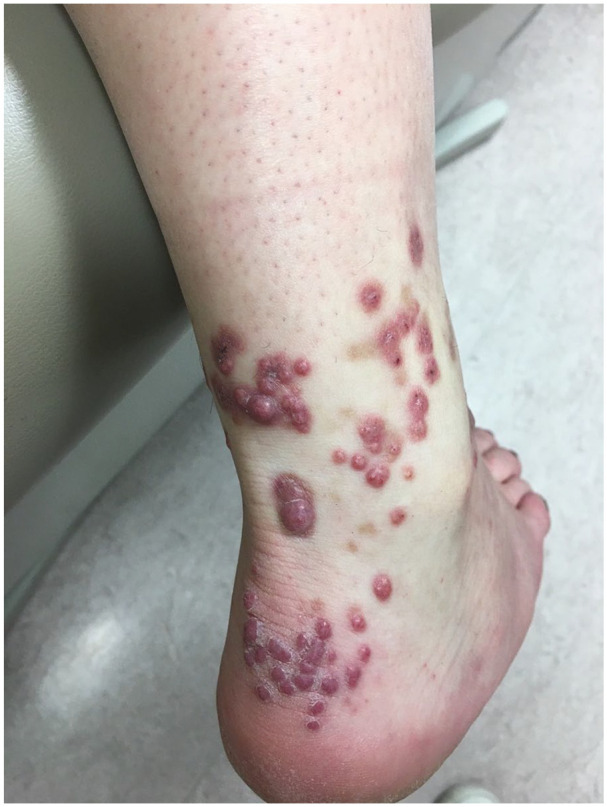
Multiple erythematous to violaceous, firm dermal papules on the Achilles and lateral ankle region of the right lower extremity.

The patient was initially treated with topical therapy alone as she was undergoing a course of fertility treatments. A super potent topical corticosteroid (clobetasol propionate 0.05% ointment) and topical dapsone resulted in little improvement. Intralesional triamcinolone acetonide 5–10 mg/ml resulted in partial resolution of some nodules, however, the patient continued to develop new nodular lesions over time (Figure 3). Systemic treatment with dapsone was ultimately initiated, however, this was discontinued within 2 weeks because of intolerable gastrointestinal side effects and transaminitis, both of which resolved with cessation of the drug. Over the next 2 years, the recurrent episodes of purpura resolved, and the patient did not develop any new nodules. Regular intralesional triamcinolone acetonide injections resulted in almost complete resolution of the remaining nodules.

## Discussion

EED is a distinct and rare form of chronic, recurrent LCV. Since its first description in 1888 by Jonathan Hutchinson, only several 100 cases have been described in the literature.^1^ The condition typically presents with firm, smooth erythematous to violaceous papules or nodules symmetrically distributed over the extensor surfaces, such as the dorsal hands, Achilles tendons, knees, elbows, as well as the buttock area.^2^ Other sites of involvement and different morphologic presentations such as ulceration, vesicles, and bullae have been described but are far less common.^3–5^ The pathophysiology of EED is characterized by immune complex deposition in vessel walls which is thought to be operative and potentially explains some of EEDs’ many systemic disease associations.^6^ These include various infectious, autoimmune, hematologic, and inflammatory disorders as well as malignancy.^7^ EED typically runs a chronic, relapsing, and remitting course with spontaneous remission ultimately occurring after an average of 5–10 years.

EED often presents a diagnostic challenge for clinicians as it typically bears little resemblance to cutaneous vasculitis. Rather, the plaques and/or nodular lesions of EED often suggest diverse entities such as dermatofibromas, granuloma annulare, Kaposi sarcoma, rheumatoid nodules, reticulohistiocytoma, necrobiotic xanthogranuloma, keloidal scarring, and bacillary angiomatosis.^7,8^ The histopathology of EED may also be misleading, as there is significant variation depending on the age of the lesion sampled. Histopathologically, early lesions of EED are indistinguishable from other neutrophilic dermatoses such as Sweet’s syndrome, Behçet’s disease, rheumatoid neutrophilic dermatosis, and bowel-associated dermatosis, and the distinction between these entities is based primarily on clinical grounds.^8–10^ The storiform pattern of fibrosis in late lesions, particularly sclerotic EED lesions, may appear similar to sclerosing hemangioma, dermatofibroma, and dermatofibrosarcoma protuberans if the biopsy does not reveal foci of LCV. Ultimately, the diagnosis of EED is based on both clinical and histopathological findings.

The present case is unusual in that classic features of recurrent LCV appeared for 2 years prior to the concurrent development of typical lesions of EED. Episodes of purpura and the pseudotumors of EED then resolved gradually over the subsequent 2 years in the same order of their appearance. This presentation allowed for a more timely and confident clinical diagnosis for our case, which was then easily confirmed with tissue sampling from both an area of palpable purpura and an older violaceous nodule. Recurrent LCV and EED both result from type 3 hypersensitivity reactions to various antigens, resulting in immune complex deposition in vessel walls, yet most patients with recurrent LCV never develop lesions of EED, while those with EED have “subclinical recurrent small vessel vasculitis” with the de novo appearance of papulonodular lesions.^9,11,12^ This may relate to differences in the inflammatory response elicited by damaged vascular endothelial cells. IL-8 is a profibrotic cytokine that is felt to play a role in several fibrotic disorders, such as systemic sclerosis, fibrosing breast cancer, as well as EED. Kimura et al. demonstrated that both serum levels of IL-8 and IL-8-positive perivascular cells were much higher in a patient with EED compared to other patients with skin-localized IgA vasculitis and cutaneous polyarteritis nodosa.^13^ A more robust IL-8 response, therefore, might explain the phenotypic differences between recurrent LCV and EED.

In summary, palpable purpura, considered by many to be the “sine qua non” of cutaneous LCV, may be an early solitary presenting feature of EED. This presentation alters how EED may be defined and widens the differential diagnosis of palpable purpura to include EED as a possible consideration. Future research into the role of IL-8 in EED may help clarify its underlying pathogenesis and aid in the development of more disease-specific therapy for fibrosing vasculitides.
